# Why We Fail at Heart Failure: Lymphatic Insufficiency Is Disregarded

**DOI:** 10.7759/cureus.8930

**Published:** 2020-06-30

**Authors:** Philip Houck, Hari Dandapantula, Evan Hardegree, Janet Massey

**Affiliations:** 1 Medicine/Cardiology, Baylor Scott & White Health, Temple, USA; 2 Medicine/Cardiology, Texas A&M Health Sciences Center, Temple, USA; 3 Family Medicine/Lymphology, Praxis Dr. Jungkunz, Friedberg, DEU

**Keywords:** heart failure, lymphatics, mortality, co-morbidity

## Abstract

Is the definition of heart failure too narrow, not allowing research into compensatory mechanisms, comorbidities, right heart function, and lymphatic function?

A review of the absolute mortality of heart failure drugs and devices suggests a modest improvement in outcomes. Absolute mortality from common comorbidities, including renal insufficiency, arrhythmia, conduction deficits, pulmonary hypertension, anemia, obstructive sleep apnea, infection, inflammation, edema, ischemic heart disease, and diabetes II, is significant. The lymphatic function is involved in short, intermediate, and long-term compensation for a failing heart and plays a role in most of the comorbidities.

A better definition of heart failure is: Heart failure is a complex clinical syndrome that results from any structural or functional impairment of right or left ventricular filling or ejection of blood and failure of peripheral compensatory mechanisms. Lymphatic function from the anatomic, fluid management, immune modification standpoints requires study. New therapies from this analysis will improve patients with congestive heart failure.

## Introduction and background

**Figure 1:**
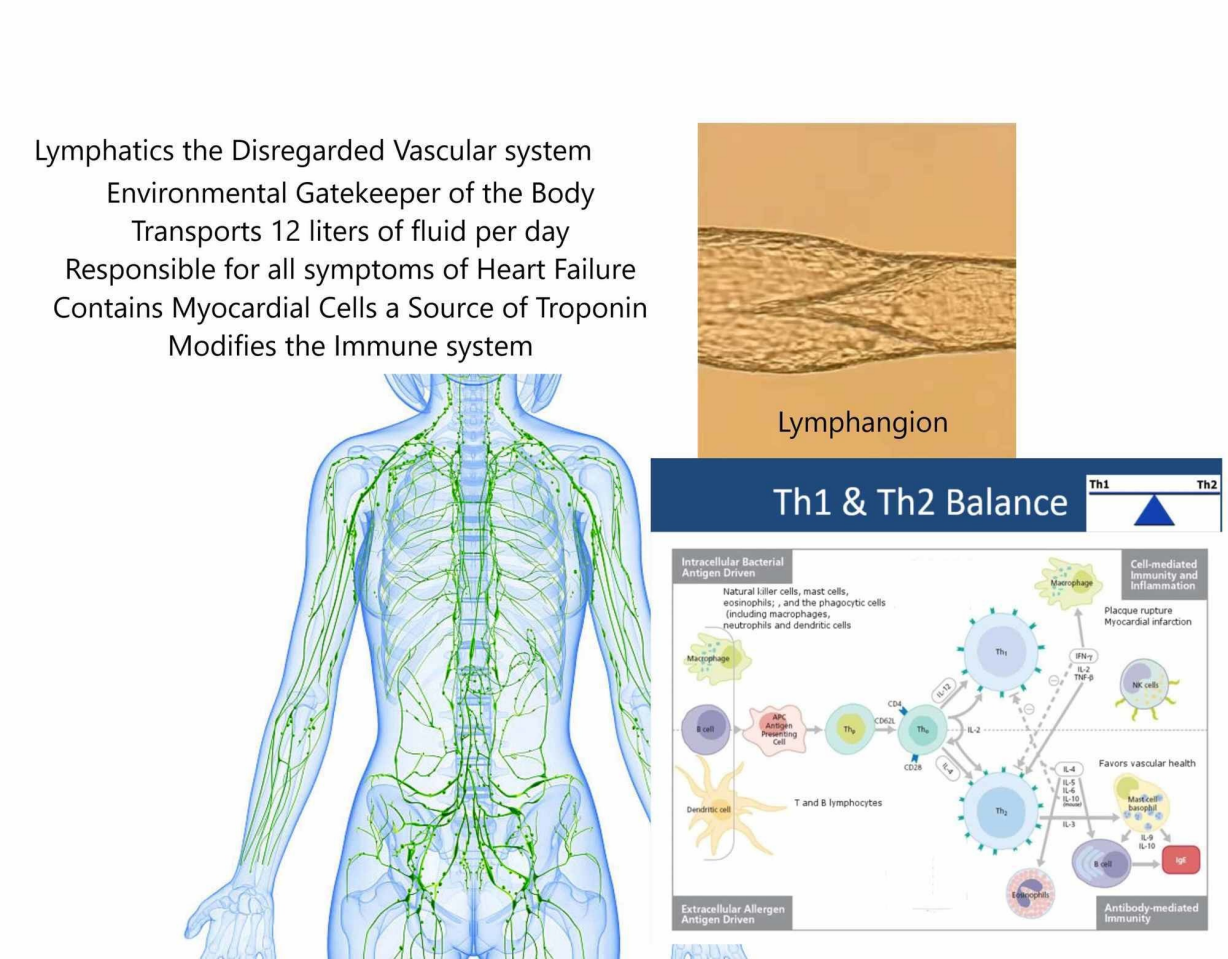
Central Illustration: The Forgotten Vascular System – Lymphatics The definition of heart failure is too narrow. Right heart function is ignored as a target of therapy. The lymphatic vascular system is responsible for heart failure symptoms and inflammation. Peripheral compensation and treatment of comorbidities represent new targets in heart failure.

Figure 1 illustrates the lymphatic system.

Heart failure is a complex clinical syndrome that results from any structural or functional impairment of ventricular filling or ejection of blood [1]. Central cardiac parameters include contractility (a systolic measure), compliance (a diastolic measure), preload (the stretch of the myocardial fibers due to filling pressures), and afterload (the resistance of flow primarily represented by the size of the arterioles). These four parameters are central factors that apply directly to the heart. The geometrical shapes of the heart and conduction synchrony are newer central cardiac performance parameters that can alter performance significantly by causing mitral regurgitation and loss of ejection efficiency.

The normal heart can accommodate a large range of pumping function from sleep, with low blood pressure and low cardiac output, to high pressure and high cardiac output during prolonged and extreme exercise. This range of performance is impressive and can rapidly change to accommodate the needs of the body by altering heart rate and venous and arterial tone. These adaptations occur nearly instantaneously. On an intermediate time scale, the body uses homeostatic mechanisms to adjust the volume status. These homeostatic mechanisms include renal function, lymphatic function, blood vessel properties, neuroendocrine system, and the autonomic nervous system. Other intermediate time-scale compensations include the musculoskeletal, hematopoietic, respiratory, and endocrine systems, and, in fact, all major organ systems and regulators of homeostasis. The intermediate compensations may take hours, days, or even months to adjust for a failing heart. Compensation on the longest time scale includes processes that remodel the structure of the heart. These mechanisms include cellular repair, hypertrophy, and fibrosis. The remodeling can be either positive or negative, allowing patient improvement or a steady decline. These processes take months to years to change the structure of the heart. Compensation in heart failure thus utilizes multiple peripheral mechanisms and varying time scales to help a structurally failing heart.

As per the 2013 American College of Cardiology Foundation/American Heart Association (ACCF/AHA) Guideline for the Management of Heart Failure: “Heart Failure is a complex clinical syndrome that results from any structural or functional impairment of ventricular filling or ejection of blood” [1]. The current definition of heart failure does not include the peripheral adaptations and compensations of the body or provide for cardiac remodeling. Comorbidities limiting these compensatory mechanisms have a significant influence on heart failure affecting instantaneous, intermediate, and long-term compensation. The heart failure population is pleomorphic in these parameters, and the definition of heart failure should include the influences of compensatory mechanisms and comorbid conditions that limit compensation. In addition, the length of current heart failure trials is insufficient to demonstrate structural remodeling compensation.

The current definition of heart failure does not acknowledge there are two hearts. A better definition of heart failure is: heart failure is a complex clinical syndrome that results from any structural or functional impairment of right or left ventricular filling or ejection of blood and failure of peripheral compensatory mechanisms. The right heart should be included in the definition of heart failure. The right heart is a thin-walled, trabeculated crescent chamber that normally operates under low pressure. The left heart is a thick-walled ellipsoid ventricle that pumps into systemic high pressure. They share the interventricular septum. The septum under normal operation supports the left ventricle. Under conditions of elevated pulmonary pressure or electrical conduction abnormalities, it supports right ventricular function. The right heart and left heart pump in series. Failure of one results in the failure of both. Right heart failure due to left heart failure is a critical risk for mortality.

Embryological development indicates that the right and left ventricles arise from two separate origins. The left heart arises from the heart tube while the right heart may derive, at least in part, from the anterior heart field (the pharyngeal mesoderm) [2]. The different origin suggests that myocardial cells may respond differently to medications. Because right heart failure ultimately determines mortality, it is necessary to target medications that affect the central right heart function and the peripheral pulmonary vasculature. The paper "Evolution of heart failure: Crotchety old cardiologist" attempted to address this knowledge gap [3].

The classes of drugs used to improve central heart failure parameters overlap peripheral compensatory factors. Neuroendocrine medications, such as angiotensin-converting enzyme inhibitors (ACEI), angiotensin receptor blockers (ARB), and neprilysin inhibitors (NI), also affect afterload. Medications that enhance lymphatic function include Nesiritide, phosphodiesterase inhibitors, sympathomimetics, and digoxin. Phosphodiesterase inhibitors, sympathomimetics, and digoxin alter contractility and preload through the enhancement of lymphatic function. Nesiritide affects both preload and afterload and compliance in addition to enhancing lymphatic function. The lymphatic function also influences the immune system, which is a major contributor to cardiac decompensation and non-cardiac comorbidities through inflammatory pathways. Medications altering the properties of blood vessels, such as spironolactone, ACEI, ARB, NI, and hydralazine/isosorbide dinitrate influence compliance and afterload. It is impossible to separate the benefits of these therapies in effects upon central parameters versus peripheral compensation. Improving peripheral compensation may be more important than the effect on central parameters. Drugs designed to improve peripheral compensation can have a significant effect on the morbidity and mortality of heart failure. In turn, comorbidities can worsen heart failure by a reduction in compensatory mechanisms.

The comorbidities are renal insufficiency, arrhythmia and conduction deficits, pulmonary hypertension, anemia, obstructive sleep apnea, infection, inflammation, lymphatic dysfunction, edema, ischemic heart disease, ischemic mitral regurgitation, and diabetes II.

Advanced heart failure specialists and pulmonary hypertension experts often do not consider the pericardium in advanced left and right heart failure. If either ventricle dilates to the limit of the pericardium, the diastolic pressures become equalized and physiology is like constrictive pericarditis. The septum demonstrates interventricular dependence - the Bernheim and the reverse Bernheim phenomena [4]. The physical exam finding of Kussmaul’s jugular venous rise with inspiration can be masked by central venous pressure being so high that the top of the column cannot be visualized. Pulmonary doctors may then blame pulmonary hypertension on left heart failure and advanced heart failure may infer volume overload instead of right heart failure. In either case, the wrong therapy may be delivered.

In the setting of advanced heart failure, hypotension, renal insufficiency, anasarca, and ascites are manifestations of right heart failure. Beta-blockers used for the treatment of left heart failure are now counterproductive. The negative inotropic effects of beta-blockers further reduce right ventricular function. Most advanced heart failure patients are restrictive in diastolic filling and require a greater heart rate to empty the already full left ventricle. Increasing the heart rate with better emptying of the ventricle can reduce functional mitral regurgitation by decreasing ventricular volumes. Heart rate is a compensatory mechanism. A failing right heart and restrictive left heart require new therapies. In some cases, medications aimed at increasing right ventricular contractility and peripheral pulmonary vasodilation may be more beneficial than negative inotropes such as beta-blockers. Including the right and left heart in the definition of heart failure will encourage future investigations leading to new therapies.

The musculoskeletal system, hematopoietic system, autonomic system, respiratory system, endocrine system, and regulators of homeostasis all have roles to play in peripheral compensation. Exercise and physical fitness have demonstrated that enhancing the musculoskeletal system leads to better outcomes in all disease processes. Exercise improves positive remodeling and takes time to accomplish. The means of compensation include increasing circulating stem cells and shifting the immune system to repair from degeneration. The compensatory enhancing intervention of cardiac rehabilitation has positive mortality and quality of life outcomes.

The respiratory system has been an enigma in therapy. One of the major respiratory comorbidities is obstructive sleep apnea with a 25.6% absolute risk of mortality [5]. Central apnea is also a common manifestation of heart failure. The enigma is that treating both apneas gives different results. Servo-controlled continuous positive airway pressure (CPAP) was studied for Cheyne-Stokes respiration. This centrally mediated apnea has high morbidity, and it makes sense that correcting this disorder should lead to an improvement. However, in the advanced heart failure patient, this type of central mediated apneic breathing is a peripheral compensatory mechanism that saves respiratory energy. Interfering with this compensatory mechanism increases mortality [6-7]. CPAP for obstructed sleep apnea should not be a contraindication in heart failure since obstruction is not a compensatory mechanism but a broken part.

Tables 1-2 illustrate cardiac performance parameters and therapeutic options with absolute mortality determining the harm or benefit of the therapy. Table 3 quantifies the net effect of comorbid conditions on heart failure absolute mortality. The causes of the comorbid condition and therapeutic options are listed and demonstrate that obstructive sleep apnea has the highest absolute mortality and all heart failure patients should be screened for obstructive apnea.

**Table 1:** Central acting cardiac performance parameters A: ACEI/ARB, B: beta-blockers, S: spironolactone ACEI: angiotensin-converting enzyme inhibitors; ARB: angiotensin receptor blocker; CPAP: continuous positive airway pressure; Bi-V: biventricular; CABG: coronary artery bypass grafting; MVR: mitral valve replacement

Cardiac Performance Parameter	Therapeutic Options to Improve Heart Failure	Absolute Mortality Benefit Harm (-)	Reference
Preload	Diuretics	-8%	[8]
	Nitrates	Unknown	
	Ultra-filtration	-8%	[9,10]
	adaptive servo-ventilation CPAP	-3.3%	[6,7]
	Tolvaptan	0%	[11]
	Nesiritide	0%	[12]
Afterload	Hydralazine/Nitrate	4.2%	[13,14]
	ACEI/ARB	1.3%	[15]
	Nesiritide	0%	[12]
	Valsartan/Sacubitril	3.2% Over A, B, S	[16]
	adaptive servo-ventilation CPAP	-3.3%	[6,7]
Compliance	Nesiritide	0%	[12]
	Valsartan/Sacubitril	3.2% Over A, B, S	[16]
	Ranexa	3.2% Unknown	[17]
	Spironolactone	5.5%	[18]
Contractility	Digoxin	0%	[19]
	Sympathomimetics	-1.5	[20]
	Phosphodiesterase Inhibitors	-1.5	[20]
Geometry and Synchrony	Bi-V pacing	4.1%	[21]
	Surgery CABG	5.0%	[22]
	Surgery MVR	10%	[23]

**Table 2:** Peripheral acting performance parameters A: ACEI/ARB, B: beta-blockers, S: spironolactone ACEI: angiotensin-converting enzyme inhibitors; ARB: angiotensin receptor blocker

Cardiac Performance Parameter	Therapeutic Options to Improve Heart Failure	Absolute Mortality Benefit Harm (-)	Reference
Neuroendocrine	ACEI/ARB	1.3%	[15]
	Spironolactone	5.5% over A, B	[18]
	Beta-Blockers	3.6% over A	[15]
	Nesiritide	0% short term	[12]
	Valsartan/Sacubitril	3.2% over A,B,S	[16]
Properties of blood vessel agents that reduce stiffness	Spironolactone	5.5% over A,B	[18]
	Nesiritide	0% short term	[12]
	Valsartan/Sacubitril	3.2% over A,B,S	[16]
	Calorie Restriction	Data limited	[24]
Lymphatic function and Inflammation	Digoxin	-0%	[19]
	Phosphodiesterase I	-1.5%	[20]
	Sympathomimetics	-1.5%%	[20]
	Nesiritide	0%	[12]
	Valsartan/Sacubitril	3.2% over A, B, S	[16]
	Lymphedema Boots	Unknown	

**Table 3:** Comorbidities/mortality - the heart failure cause/solution Hs-CRP: high-sensitivity C-reactive protein; RV: right ventricle; LV: left ventricle; LBBB: left bundle branch block; IVCD: intraventricular conduction delay; Bi-V: biventricular; IV: intravenous: intravenous immunoglobulin

Comorbidity	Absolute Mortality (-) Harm	Ref	Cause	Solution
Renal Insufficiency % decrease per mL/m of creatinine clearance	-1%	[25]	Over diuresis	Stop diuresis
			Bladder obstruction	Bladder scan urology consult
			Neurogenic	Straight catheterization
			Males – prostrate	Green-light vaporization
			Females – pelvic floor	Straight catheterization
			Medications	Stop offending agent
			Decreased cardiac output	Increase cardiac output
			RV failure or restrictive LV	Increase heart rate
Arrhythmia conduction	-11%	[26]	Atrial fibrillation	Rate or rhythm control
	-19.4%	[27]	Ventricular tachycardia	Treat CHF – anti-arrhythmic
			Bradycardia – Heart block	Decrease blockers pacemaker
	-3.1%	[28]	LBBB/IVCD	Bi-V pacemaker/Defibrillator
Pulmonary hypertension	-25.6%	[5-7]	Obstructive sleep apnea	CPAP
			Lung disease	Optimize medications – O_2_
			Diastolic dysfunction	Medications - increase HR
			Valvular dysfunction	Valvular intervention
			Pericardial disease	Medications or intervention
Anemia	-17.3%	[29]	Iron deficiency	IV iron therapy
			Inflammation	Colchicine
			Renal insufficiency	Erythropoietin therapy
			Myelodysplasia	Erythropoietin therapy
			Testosterone deficiency	Testosterone replacement
			Vitamin deficiencies	Vitamin supplement
Infection	-.8%	[30]	Flu - viral illness	Immunizations
			Bacterial Illness	Immunizations
			Myocarditis Endocarditis	IVIG
Inflammation Hs-CRP	-32%	[31]	Abnormal immune response	Colchicine
Lymphatic dysfunction			Thoracic duct injury	Lymphedema boots
			Inhibiting medications	Discontinue offending agent
			Infection tissue injury	Treat and support
Edema	-6%	[32]	Dietary salt intake	Dietary management
			Inflammation	?
			Iatrogenic medications	Remove agents
			Lymphatic dysfunction	Lymphedema boots
Coronary disease	-28%	[33]	Recurrent myocardial infarction	Surgery; Colchicine
Ischemic mitral regurgitation	-20%	[34]	Annular dilation, LV geometry	Surgery
Diabetes II	-10.2%	[35]	Insulin excess; Lack of exercise; Excessive calories	Emplaglitizone; Exercise; Good nutrition

## Review

Models of heart failure do not incorporate major physiological systems. The lymphatic system is a major vascular system that is responsible for the compensation of cardiac dysfunction and repair of the heart and periphery. Despite the ability to transport 12 liters of fluid per day, the transport of stem cells to sites of injury, and the modification of the immune system, the lymphatic system is not considered in congestive heart failure. This system is responsible for the most common symptoms of heart failure - edema and dyspnea. The lymphatic function has a prominent role in most comorbidities that are responsible for cardiac mortality, including cardiorenal, infection, inflammation, and diabetes II. There is no systematic classification of drugs assigning positive or negative lymphangiotropy to the drug effect. Strategies to enhance lymphangiotropy should improve the quality of life by mobilizing the interstitial fluid. The paper "Alternative view of congestive heart failure exacerbations: Role of lymphatic function and inflammation" outlined this strategy [36]. Milrinone, dobutamine, Neseritide, and digoxin given for decompensated heart failure have positive lymphangiotropy. Other agents have a negative lymphangiotropy effect and can cause worsening heart failure symptoms such as calcium channel blockers and Pioglitazone.

Undiscovered lymphatic processes in heart disease

Location of Vegetation in Endocarditis

Lymphatic drainage of cardiac valves runs on the low-pressure side of the valve: the ventricular aspect of the aortic valve and the atrial aspect of the mitral valve. The lymphatic system is designed to control infection by inducing edema and decreasing systemic spread. The location of lymphatic drainage is the most plausible reason for the location of vegetation. Previous explanations invoke low sink hemodynamic swirling with endothelial damage and secondary infection of platelet fibrin nidus. Endothelial damage disrupts the infective lymphatic flow, with tissue edema. Bacteria are trapped in the dysfunctional lymphatic and grow to form the vegetation.

Troponin Leaks Post-Surgery, With Sepsis, Critical Illness - Type 2 Myocardial Infarction

The source of troponin in the above clinical syndromes is assumed to be the heart since troponin is reported to be specific only to the heart. The leak represents poor prognosis in the short term and is associated with long-term adverse cardiovascular outcomes [36]. The management of these patients is more difficult, with these patients generally being older and having worse kidney function. Cardiovascular testing and intervention is performed without warranting clinical symptoms. The outcomes of this approach are lacking [37]. The harm of these interventions may be greater than the benefit considering contrast-mediated renal disease and antiplatelet therapy in an older population with renal insufficiency. The reference by Scallan et al. suggests the lymphangion has cardiac cells that express troponin C and I as well as cardiac isoforms of tropomyosin [38-39]. In most clinical scenarios associated with a type 2 infarction, the lymphatic system is under considerable stress from infection, edema, and intravascular volume or pressure. The distressed lymphangion is a more likely source of low troponin elevations in these sick individuals. Therapy should be directed toward lymphatic salvage as opposed to cardiac intervention. It is not always the heart, but the underappreciated lymphatic vascular system that rules patient prognosis.

*Cardio-Renal Syndrome* 

The pathophysiology of the cardio-renal syndrome has been elusive. It suggests a connection between the heart and kidney. The typical scenario is the presentation of a fluid-overloaded patient secondary to heart failure. The initial creatinine is normal. The patient receives a loop diuretic with a failure of diuresis and a rise in creatinine. The diuretic is held, and symptoms of heart failure are managed by various means while awaiting improvement in creatinine so diuresis can again be attempted. If the syndrome is not managed, renal dialysis or death ensues.

Explanations of low systemic pressure or low cardiac output have not been proven. Elevated venous pressure appears to be one of the best predictors. Lymphatic drainage of the kidney is not considered. An experiment performed many years ago may be the reason lymphatic function is ignored in this syndrome. The experiment was to disrupt main lymphatic drainage from the kidney and the result was increased urine flow. Obstructing the outflow of the lymphatics will result in pressure within the glomerulus that favors tubular excretion. This condition is distinctly different from failure of the lymphangion that would increase glomerular pressure by edema formation with an unfavorable tubular flow. The hypothesis is that loop diuretics, in the setting of high venous pressures, reduces glomerular lymphangion amplitude and frequency, increasing interstitial edema of the kidney. The therapy is to provide medications that have positive lymphangiotropy, improving amplitude and frequency. The medications that accomplish this function are sympathomimetics, phosphodiesterase inhibitors, and Nesiritide, which increase lymphangion amplitude and frequency. Empagliflozin, a sodium-glucose co-transporter 2 (SGLT2) inhibitor, has great enthusiasm as a new agent to treat heart failure even in non-diabetic patients. The mechanism has not been elucidated. The ability to reduce glomerular pressure likely occurs by positive lymphangiotropy by this diabetic drug [40].

*Takotsubo* *Cardiomyopathy*

Takotsubo cardiomyopathy is a transient left and sometimes right ventricular dysfunction, which has several anatomical presentations. The pathophysiology of this disorder has not been elicited; however, it is associated with abnormally high catecholamine states. The transient left ventricular function is associated with increased wall thickness as opposed to ischemia, which thins the walls. There is the classic apical form, which can involve the left or left and right apex in a non-coronary distribution, a mid-ventricular form, and a basal form. The reasons for this noncoronary distribution of dysfunction has not been elicited. A hypothesis is that the lymphatic penetration of the heart is segmental, involving only the apex, middle, or basal segments. Acute dysfunction of one or multiple distributions will result in myocardial interstitial edema with the distribution of left ventricular function noted. Improvement in lymphatic function and resolution of the edema allows myocardial recovery.

The origin of lymphatics is unknown. If one assumes the human body is a product of evolutionary life, borrowing successful systems from its predecessors, it is likely this parallel circulatory system came from insects. Insects’ lymphatic circulation was the main mechanism for supplying hemolymph to a complex multicelled segmented life form. The system exposed to the environment also had to function as an immune gatekeeper. This system evolved more than 350 million years before the mammalian circulatory system of heart, arteries, capillaries, and veins. Speculation would suggest that this parallel circulatory system was added to the mammalian by incorporating deoxyribonucleic acid (DNA) from insect life forms - a chimera of sorts. The concept of adopting an entire system into another organism is a new and unsubstantiated concept. In terms of embryogenesis, the lymphatic system is first recognized two weeks after the appearance of the circulatory system. Although lymphatics frequently run in the same direction as arteries and veins, they appear to be independent in development and segmented. The lymphatic system is in every organ but is concentrated in the lung, gastrointestinal tract, and skin where the environment interacts with the mammalian organism.

Relationship of inflammation and heart failure

C-reactive protein (CRP) is a measure of the response of the immune system and has demonstrated clinical significance in myocardial infarction. In heart failure, CRP has a similar relationship. The chicken or egg debate exists as to whether inflammation is caused by heart failure due to poor perfusion of the gut, resulting in microbiome translocation or if inflammation decreases, lymphatic function with a decrease in peripheral lymphatic compensation. CRP is elevated when brain natriuretic peptide (BNP) is elevated. The elevation of CRP is associated with reduced function of the lymphangion. Inflammatory states cause negative lymphangiotropy, with a reduction in the amplitude and frequency of contraction. Injury begets swelling, and systemic inflammation begets heart failure. Diabetes II, influenza, infections, sepsis, and clotting are all inflammatory conditions and frequently coexist with heart failure. Treating inflammation in heart failure seems to be as reasonable a target as myocardial infarction.

*Education into practice* 

Heart failure trials do not include compensatory mechanisms. Pharmacologic and pacing strategies for right heart failure are lacking. The accepted definition of heart failure does not include intermediate and long-term compensatory adaptations of non-cardiac peripheral systems. Amending the definition of heart failure to include peripheral compensatory mechanisms and right heart physiology could identify novel targets for investigation and ultimately aid in improving patient outcomes. The entire lymphatic vascular system has been disregarded. Dr. Massey would suggest that the term chronic lymphatic Insufficiency should be incorporated into the definition of heart failure. 

The new definition of heart failure suggests the following areas of investigation:

Pharmacologic and mechanical enhancement of the lymphatic system to improve heart failure compensation

Pharmacologic and mechanical enhancement of the immune system to reduce inflammation and promote repair rather than fibrosis

Lymphatic function in comorbid conditions, cardiorenal, Takotsubo cardiomyopathy, infections, type 2 myocardial infarction, diabetes, and renal insufficiency need further investigation. 

The comorbidities of study populations need meticulous definition and changes in these comorbidities secondary to drugs or devices should be separate endpoints. In other words, did the intervention change the comorbidity, and did this change have a beneficial result on primary endpoints.

Directing therapies toward renal protection, modifying the immune system (high-sensitivity C-reactive protein; Hs-CRP), diagnosing and treating obstructive sleep apnea, anemia, and diabetes II can have a significant impact on heart failure mortalities. This impact may be greater than the guideline-directed medical therapies listed in Tables 1-2.

Heart failure trials need to be longer in duration to establish structural changes in the heart due to therapies.

## Conclusions

The new definition of heart failure that includes compensatory mechanisms and investigates both the left and right heart will lead to new strategies and new therapies, extending the scope of therapy beyond the heart. The new definitions will boost research in lymphatic function. The new definition of heart failure should be “Heart failure is a complex clinical syndrome that results from any structural or functional impairment of right or left ventricular filling or ejection of blood and failure of peripheral compensatory mechanisms.”
